# Making sense together: participatory sensemaking, learning cycles, and group roles

**DOI:** 10.3389/fpsyg.2026.1746763

**Published:** 2026-02-23

**Authors:** Christian Kronsted, Matthew Henley, Miriam Giguere

**Affiliations:** 1 The Honors Program, Merrimack College, North Andover, MA, United States; 2 Dance Education, Teachers College Columbia University, New York, NY, United States; 3 Department of Performing Arts, Drexel University, Philadelphia, PA, United States

**Keywords:** affordances, ecological psychology, embodied cognition, group learning, group roles, Kolb, learning cycle, participatory sensemaking

## Abstract

The Kolb Learning Cycle is a popular model of experiential learning in which agents move through four phases: experimentation, concretization, observation, and conceptualization. This model is a dynamic learning model that aligns well with embodied approaches to cognition, as it centers on student agency, inquiry, and exploration. However, there is currently no 4E (embodied, enactive, embedded, and extended) account of the learning cycle. Furthermore, Kolb’s theory focuses solely on behavior and learning in the individual. We here create a 4E account of the Kolb learning cycle by combining it with group role theory, ecological psychology, and participatory sense-making (PSM). We argue that, as individual members cycle through various group roles and their associated Kolb phases, they aid the group as a joint cognitive system in transitioning to new modes of engagement at the group level. Moving through group roles (leader, follower, naysayer, observer) often moves the agent into a new Kolb phase, which, in turn, changes the emergent dynamics of the entire group. Thus, social interaction can drive the learning cycle. Because the behavior of the individual is emergent, we cannot rely on reductivist accounts to explain group learning behaviors as the outcome of individual contributions. Rather, we consider the group as a cognitive system that drives learning.

## 1 Introduction

In this paper, we begin from our combined experience as researchers and dance educators. While facilitating creative group processes, such as the development of collaborative choreography, we observed that the students’ processes and products were often unexpected, unique, and dependent on the makeup of the group—that what students learned depended on who they were learning with. Though there are a number of theories that provide an explanation for aspects of these observations—i.e., group cognition, embodied cognition, participatory sensemaking, Kolb’s learning theory—each one taken individually does not fully account for our observations. In this paper, therefore, we bring these theories together. We begin by outlining dynamic systems research in cognitive science. Next, we provide an overview of the embodied cognition framework of participatory sensemaking. These frameworks are explicitly about cognition and only implicitly about learning. We, therefore, bring these two frameworks explicitly into the learning sciences by combining them with Kolb’s theory of learning cycles. Taken together, this conceptual integration contributes to the literature by providing a theoretical framework for further empirical study into how learning occurs during complex creative group processes.

Classical accounts of thinking and its developmental counterpart, learning, are often framed as individual, internal, linear, and hierarchical processes. A variety of research agendas have analyzed group cognition and learning. However, these approaches start from the individualistic perspective and then move to a collective account (Bratman, 2014; Searle, 1990; Knoblich et al., 2011; Jankovic and Ludwig, 2017; Gilbert, 2020). The growing research field of 4E cognition reverses this direction and argues that cognition is inherently social, and from the social, one becomes an individual (Newen et al., 2018; Hutto, 2008; Hipólito et al., 2021; Di Paolo et al., 2018; Weichold and Thonhauser, 2020). What we observe as artists and educators fits hand in glove with the 4E perspective; groups seem to have a life of their own and individual students behave differently depending on the makeup of the group. Therefore, the conclusions that might be drawn about an individual in one context might not transfer to the same individual in another context. If we prioritize individuals as the unit of analysis, we miss something about what the group as a whole is doing. As the science of non-linear dynamics has shown us; group behavior is not reducible to the sum of its parts (Camazine et al., 2020).

When looking at what groups are doing as whole entities, one particular approach lends itself well to this form of analysis—namely, dynamic systems theory (Dale, 2024). Dynamic systems is a mathematical approach to understanding cognition that takes the organism’s environmental coupling as its unit of analysis and does not rely on assumptions about internal brain-bound representation-based computation. From the perspective of dynamic systems, groups are coherent, cohesive, agential systems with unique behavioral profiles we can map, test, and predict (Warren, 2018; Favela and Chemero, 2023). In recent years, participatory sensemaking (PSM) has become a leading theory of how human learning and language occur as a coupled and emergent system in dyads or groups (De Jaegher and Di Paolo, 2007; Di Paolo et al., 2018; Cuffari and Figueiredo, 2025). PSM reframes the starting point of cognition to the social encounter between agents (De Jaegher et al., 2010).

In preparation for future empirical studies, we look at the intersection of dynamic systems (Dale, 2024), Participatory Sense-Making (De Jaegher and Di Paolo, 2007), and Kolb Learning Theory (2014). The Kolb Learning Cycle is a popular model of experiential learning that outlines concept development through four phases; experimentation, concretization, observation, and conceptualization. Kolb’s approach to experiential learning provides a framework to look closely at the moments of interaction involved in PSM. We focus on Kolb specifically because it is a dynamic learning model that centers student agency, inquiry, and exploration. In other words, Kolb’s Learning Cycle is part of what has been opaquely named the “active learning” tradition (Cunha et al., 2024). Rather than being a model of optimizing passive learning environments, the learning cycle demonstrates how learning can be achieved while students simultaneously become engaged, motivated, agentive, and independent learners. The Kolb cycle shows that the acquisition of new skills and knowledge is a dynamic and active process and, therefore, non-linear and interaction-based. The model, therefore, shares many characteristics that lend itself to integration with the 4E sciences.

Although there is much research on the cognitive behaviors of complex systems, much of this research is conducted in laboratory settings, which raises concerns about the ecological validity of claims about emergence. We, therefore, situate our conceptual analysis in the context of real-world agents engaged in a real-world process, i.e., students learning how to create dances that explore personally meaningful concerns. As an example of the productivity of these combined theoretical perspectives, we merge dynamic systems and PSM with Kolb’s learning cycle considering how cognition might be embedded in and distributed across a group of individuals engaged in a shared task. We argue that integrating dynamic systems and PSM with Kolb’s Learning Cycle provides a relevant and specific framework for understanding learning as a dynamic, non-linear, and emergent process. Emerging from the combination of these theoretical perspectives, we later propose several areas for further empirical study, including the possibilities that:

1. Both individual and group cognition are non-linear and emergent.
2. Group cognitive activity is partially constituted by interactions between members and therefore cannot be reduced only to the behaviors of individuals.
3. Learning in group contexts is a non-linear cyclical process that involves individual members taking on different roles.
4. As individual members cycle through various group roles with their associated Kolb phases, those members aid in pushing the group as a joint cognitive system through phase transitions into new modes of engagement at the group level.

Throughout the analysis, we will exemplify our claims by referencing our professional experience, supported by relevant literature, with facilitating collaborative dancemaking as a productive site for examining group thinking from a 4E perspective.

## 2 Dynamic systems and affordances

Since the mid-20th century there has been a slowly accelerating embodied turn in the cognitive sciences—moving away from the cognitivist computer metaphor of the mind toward a model of cognition and perception being tied to embodiment and action (Newen et al., 2018). Though there are a variety of, often competing, theories, in general, cognition is framed as the process of an organism maintaining dynamic equilibrium in social and material environments. The human organism is unique in the complexity of its sociomaterial niche and therefore requires complex explanatory frameworks.

First, we must understand a little bit more about non-representational approaches to cognition or what is often called “radical” approaches to cognition—enactivism, ecological psychology, dynamic systems theory, and embodied cognition more broadly (Meyer and Brancazio, 2023). Dynamic systems approaches model cognition not as individual brain-bound processing of mental representations but rather as something that the entire organism achieves while engaging with its environment (Dale, 2024; Kelso, 2021). Ontologically speaking, “cognition” is the name we give to certain kinds of adaptive biological processes over time as precarious organisms attempt to stay in equilibrium with their environments (Beer and Di Paolo, 2023). We model such interactions between agents and the environment through the mathematics and language of non-linear dynamic mathematical differential equations (Beer, 2023). In short, dynamic systems approaches to cognition model cognition as the emergent behavior of coupled systems over time (Fuchs, 2018; Gallagher, 2020). Rather than the linearity of the traditional “information processing” model (i.e., input, decision, output), such systems continually move through landscapes of attractors, repellers, saddle points, stable and meta-stable cycles, and more. The use of nonlinear dynamics models shows that agents and environments co-constitute each other as they mutually change over time (Di Paolo et al., 2018).

Rather than bodily action merely influencing cognition (which is the view of standard computational cognitive science), in embodied cognition, the interactions are the cognition. In other words, cognition is a collection of relational real-world processes. Constitution versus causation is a large debate within cognitive science. However, it is far outside the scope of this paper to address that debate here (for some examples, see; Aizawa, 2010; Alexander, 2025).

Cognition takes place as agents and environments are coupled through what are known as affordances. One prominent understanding is that affordances are relations between agents and environments that provide possibilities for actions (Chemero, 2009; Gibson, 1979; Wilkinson and Chemero, 2024). For example, a keyboard affords typing, a racket affords holding, a chair affords sitting, a button affords pushing, pants afford wearing, stairs afford walking, and so on. These relations exist between the agent and the environment as the agent is coupled with the environment. While some of the coupling is mechanical coupling (as in the examples above), many of the ways we are coupled to the environment happen through the ambient energy array; the field of light, sound, heat, vibration, and so on, which carries covariant ecological information (Chemero, 2009; Favela, 2024). The upshot of coupling through the ambient array is that affordance theory is a theory of direct perception (Gibson, 1979). Agents do not need to create mental models of the world “inside” their minds because they are already engaging directly with the world (Brooks, 1991).

As agents move through the world, they are constantly presented with a complex and changing field of affordances upon which to act (van Dijk and Rietveld, 2017). For each individual agent, there exists a unique field of relevant affordances (Rietveld and Kiverstein, 2014) shaped by personal dispositions and cultural practices. With each action emerges new affordances in an ongoing action-perception cycle. Affordances ebb and flow, morph and change, with various factors, including the agent’s skills, state of the body (fatigue, hunger, thirst, etc.), contextual demands, and more. Only intellectually can we separate perception from action (Gallagher, 2017). We can say that agents follow various “lines of affordances” (Kronsted, 2024) as they are based not only on the agent’s body but especially on their skillful habits (Bruineberg et al., 2021) and the way those habits are shaped by cultural values. Cultural contextual demands often include covert or overt social norms, specific cultural activities, social rituals, seasonal norms, and the list goes on (Kiverstein, 2023). As agents follow affordances and interact with the world, they become more skillful, and their affordances, in turn, become more nuanced. Embodied habitual skill brings out more nuanced affordances that further allow the system to become more adaptive in increasingly complex and nuanced contexts (Di Paolo et al., 2018), contributing to both enskillment and enculturation.

On such an account, knowledge is understood as dispositional available skills (Kronsted et al., 2023). To know how to do quadratic equations is to act correctly, according to the norms of mathematical practice, when in the presence of certain kinds of mathematical symbols (Kronsted and Gabriella, 2025). An agent knows something when they have the skill to act in a certain way that fulfills the material and/or cultural demands of the context. The agent is dispositioned to act in particular patterns given particular available affordances (Di Paolo et al., 2018; Seifert et al., 2021). Every time an agent acts in the world, they are asserting their dispositional skills and simultaneously fine-tuning those skills in relation to the demands of the various contexts. Even abstract symbol manipulation, which is often labelled the hallmark of human cognition, remains rooted in dispositional skills (Monterroza-Rios and Gutiérrez-Aguilar, 2022).

Taken together, an affordance-based dynamic systems approach suggests that a theory of cognition must account for the fact that humans are constantly grappling with multiple nested opportunities for interaction. And that over time, enskillment and enculturation are the result of honing and refining those interactions. In the next section of this paper we turn our attention to the theory of Participatory Sensemaking, which explicitly addresses the social dimensions of an affordance-based dynamic systems approach.

## 3 Participatory sensemaking

Participatory Sensemaking (PSM) is a non-representational theory of social cognition that outlines how embodied agents make meaning together (De Jaegher and Di Paolo, 2007). PSM also utilizes the notion of affordances (Gibson, 1979), focusing on social affordances, which are possibilities for actions that exist as relations between an agent and other agents. As described above, these relations exist at the nexus of each agent’s embodied skills, social and cultural history, upbringing, emotional and affective states, gender and racial identities, and a myriad of other factors (Brancazio, 2020; Wilkinson and Chemero, 2024; Fuchs and Koch, 2014; Seifert et al., 2021; Dings, 2019). In this model, meaning is co-created as agents become coupled into a joint system and jointly regulate their coupling. Cognition and meaning are one and the same process that happens in whole systems across agents.

In PSM, each agent takes turns directly regulating the embodied system of the other agent through the creation of social affordances. Classically, in everyday interactions, the outstretched hand provides the social affordance of “shake-ability” and “interaction start.” In dance making, showing a collaborator a potential movement for a choreography affords the partner to copy that movement and add their own input to what should come next in the sequence. Each agent takes turns, acting on social affordances and thereby providing social affordances back again in an ongoing loop. With each action, each agent, in turn, creates new social affordances for the other agent. Through the process of directly regulating each other, each agent also perpetuates and regulates their coupling and thereby perpetuates the overall system (De Jaegher and Di Paolo, 2007).

We see, in this theory, the importance of embodied synchronization. With each acting and passing of social affordances, the agents become more synchronized with each other and thereby also more attuned to one another. With higher attunement comes more nested ecological information, thereby creating a loop of more and more meaningful social interaction. A wealth of empirical literature demonstrates that when human beings interact and create meaning together, they increase their synchronization across nodding, gestures, posture, breathing, gaze patterns, gait patterns, brainwave synchronization, and the list goes on (Ayache et al., 2021; Dikker et al., 2017; Schilbach et al., 2013; Abney et al., 2021). This increase in synchronization, in turn, makes the social affordances provided between agents nested with more information and makes the exchange of affordances smoother and even more meaningful (Seifert et al., 2021). There is a causal relationship between bodily synchronization and affordance production in which the two perpetuate and strengthen each other. Here, think of the way that long-term romantic partners can signal a wealth of information to one another through simple gestures. Those same gestures mean very little to people outside the relationship. The history of interaction builds the possibility for more nuanced and meaningful interaction. Similarly, group members working together build long-term attunement that allows for more complex social interactions.

In this view, social systems are metastable and non-linear and must therefore be actively perpetuated. In a conversation, if one person does not answer a question or lets their attention drift, the conversation dies. In perpetuating the coupling between agents, the system as a whole produces emergent behaviors. Such behaviors cannot be reduced to the sum of their parts but are a direct result of the ongoing nonlinear interactions between agents. Colloquially, we say things such as “Wow this is a scary conversation” or “we were all bullshitting.” Sometimes, it is apparent that a social interaction system changes from one mode to another. Through the perpetuation of the system, agents can make the system go through a phase transition and shift modes. For example, a conversation might start as playful and light-hearted, but then as conversations flow, participants end up talking about politics. In such situations, we say that “the mood changed” or “the conversation changed”, though it is possible that no one agent initiated or directed this change. As the system goes through a phase transition from one mode of interaction to another, the overall landscape of affordances available to participants also shifts.

Adopting a dynamic systems and PSM approach to human cognition frames groups as metastable emergent organizations of parts, in which the behavior of the entire system cannot be reduced to the sum of its parts—social interactions are self-organizing (Camazine et al., 2020; Tognoli et al., 2020; Kelso, 2016). As agents couple and become synchronized, the system as a whole self-organizes into emergent behaviors and modes of engagement. Such modes, in turn, determine what can be further done and said; the modes set the parameters for the continuation of the system (Gallagher, 2020). The affordances an agent can produce within a system that is in the mode of “play-fighting” is very different from the affordances available and producible within the mode of “scholarly inquiry.” Modes of engagement are developed and maintained through interactions but are also impacted by the general behavior settings of physical and cultural environments, the affect of environments, and institutional rules and regulations (Raja and Heras-Escribano, 2023; Heft, 2024; Petracca and Gallagher, 2020).

In the review above, we have seen that, according to dynamic systems theory, cognition is constituted by affordance-based agent-environment interactions. PSM demonstrates how social cognition is relationally co-constituted and that group-level agency is emergent. As researchers and dance educators interested in the application of these theories to educational environments, an important distinction emerges. Dynamic systems theory and PSM tend to focus on the moment-to-moment interactions that comprise cognitive activity, rather than the relatively stable, long-term, impact of those interactions, or what could be called learning. There is existing literature on learning from a 4E perspective (Avilés et al., 2020; Vaughan et al., 2021). This work, however, attends to the individual agent, rather than dynamic engagement within groups. In order to theorize an explicitly emergent and social theory of learning, we integrate a 4E lens, via dynamic systems and PSM, with a widely used framework for understanding learning as a dynamic and emergent process – namely Kolb’s Learning Cycle. In the next section, we provide an overview of this theory before turning our attention to integrating these frameworks.

## 4 The Kolb learning cycle

**Figure 1:**
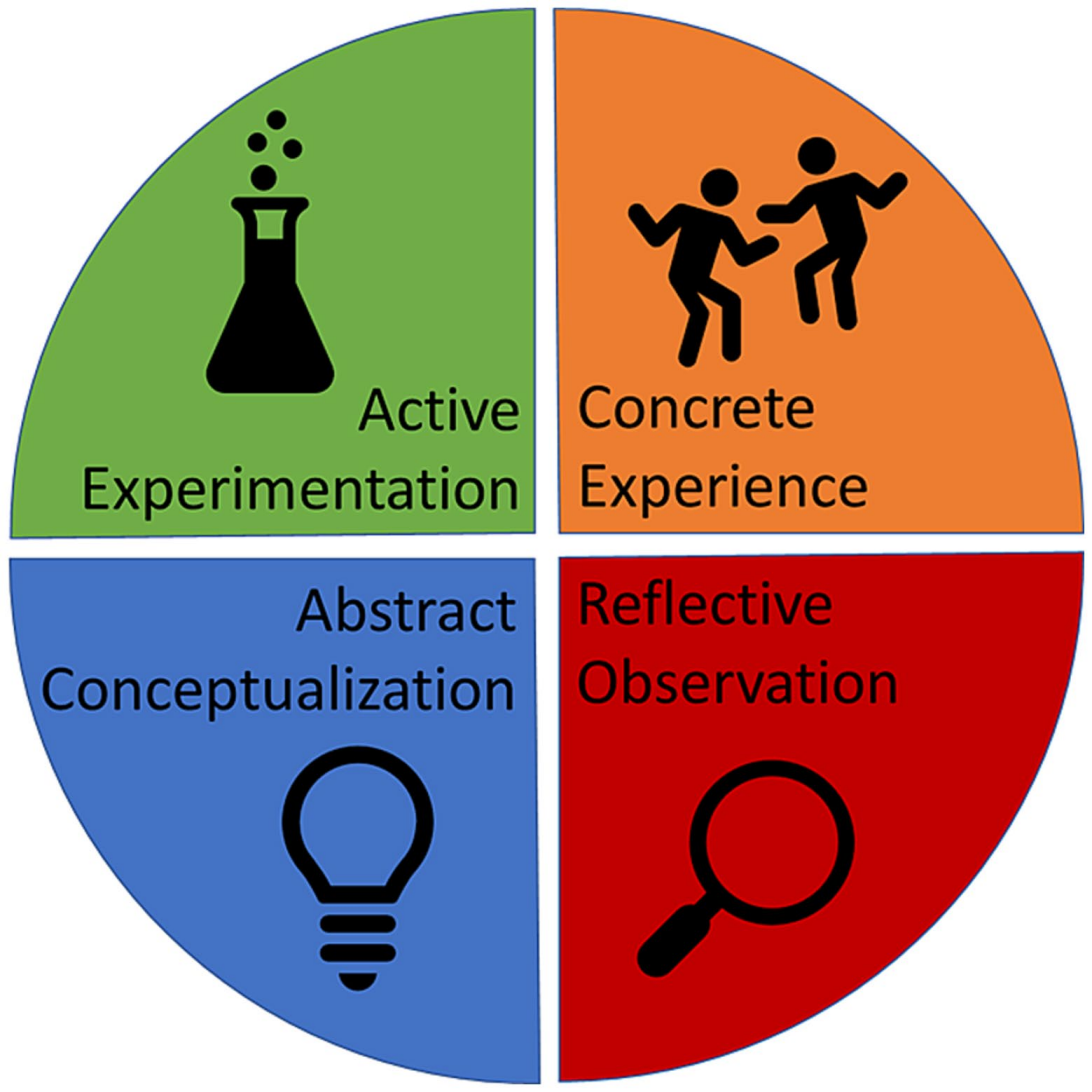
The Kolb learning cycle. The cycle can run in any direction, but typically runs clockwise, starting with concrete experience. However, the cycle can begin from any stage.

Kolb’s “Reflective Cycle” or “Experiential Learning Cycle” (Kolb, 2014; Kolb, 1984; Kolb and Kolb, 2017) is a cognitive model that outlines how agents acquire knowledge through a cyclical process of transforming experience in four stages: concrete experience, reflective observation, abstract conceptualization and active experimentation (see Figure 1).

### 4.1 Concrete experience

The learner actively engages in new experiences or re-engages an existing experience. Agents in this stage are immersed in a task and its corresponding experiences. Rather than focusing on abstract interpretations or analyses, the agent is sensor-kinetically engaging with the environment. This stage typically involves various degrees of attentional “immersion” as the agent focuses primarily on the immediate sensor-kinetic dimensions of the task at hand. From the perspective of a choreographic process, this may look like group members engaging in movement creation or physical brainstorming through improvisation. It will also be a time when members learn and practice new sequences to embody the movement suggestions of their peers.

### 4.2 Reflective observation

The agent reflects on their recent experiences, recalling, analyzing, and drawing inferences. Reflective Observation often involves “stepping back,” confirming or rejecting concepts and principles developed from previous engagements with experiences. In addition to causal, logical, cultural, and social considerations, such active processing also includes the affective and emotional components of the experience. Importantly, reflective observation often allows the agent to make sense of the experiences from different perspectives empathetically: “I experienced the task like this, but Maya probably experienced it like that.” In our dancemaking example, reflective observation may occur as group members watch one another moving, reflect on the choreographic process outside of rehearsal, or even reflect on their own process in the midst of moving. Interesting, problematic, or emotionally rich experiences begin to become apparent to the participants, although not always yet expressed to the group.

### 4.3 Abstract conceptualization

The agent now develops general principles based on the experience-grounded reflections from the previous stage. This typically involves the changing, combining, sharpening, and so forth of “abstract” concepts. In this process, new ideas, theories, and concepts are developed, and connections are made between concepts. In our dancemaking example, abstract conceptualization occurs as the world of the dance comes into focus and an “aboutness” emerges. It also occurs as group members may choose to critique one another or problem-solve specific opportunities for development, referencing experiences that are or are not present in the choreography.

### 4.4 Active experimentation

The agent now applies newly formed concepts and principles to their environment. This involves testing theories, taking action, and experimenting with different approaches. With each application of their concepts, the agent keeps track of the outcome and measures the outcome against their intentions, predictions, theories, and so on. As concepts are being tested against the real world, new possibilities for further investigation emerge, and the cycle starts over. This is where the recursive, iterative revisions of the choreography take place as corrections, additions, and revisions are tried and tested against conceptual, emotional, or aesthetic expectations.

**Figure 2:**
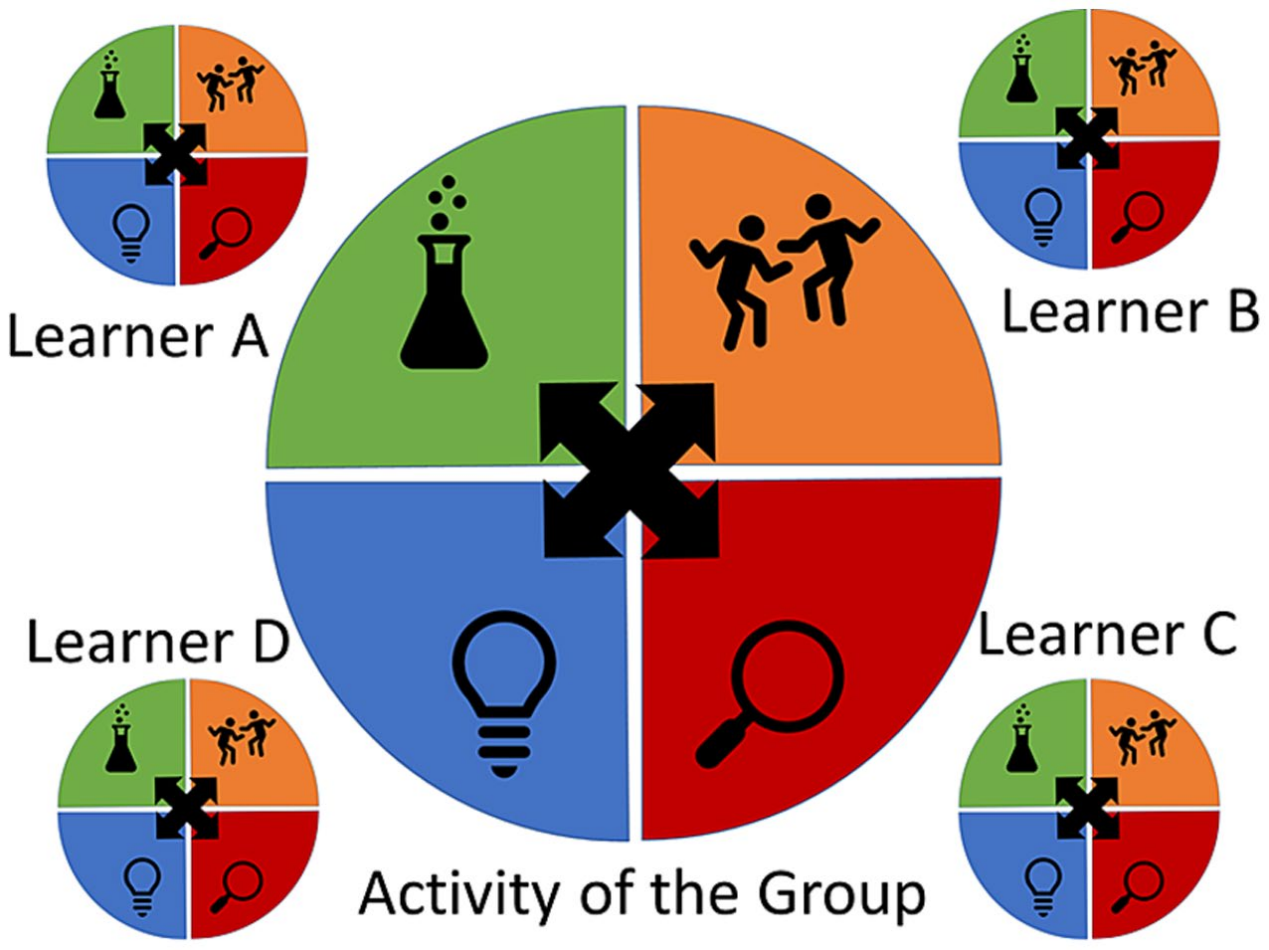
Examples of different agents moving through the learning cycle non-linearly.

According to Kolb, the agent enters into the learning cycle from any stage and will repeatedly cycle through the stages as ideas and actions are developed, experienced, and integrated. Effective learning happens at the completion of a cycle when a new concept has been experienced, reflected upon, comprehended, and introduced into action and experimentation. As complex ideas form and skilled habits are developed, the agent will cycle through the stages again and again, contributing to the type of enskillment and enculturation described above. We see then, that learning on this model, is a dynamic process that emphasizes constant experiencing and transformation of that experience. At no point in the learning cycle is the agent passive. Rather, the agent must actively grapple with the environment in various ways to achieve mastery (an important point shared with the 4E cognitive sciences). Learning and conceptualization come from an ongoing commerce with the environment through processes of experiencing, attuning, adjusting, and applying. In these ways, Kolb’s theory aligns well with a 4E lens. In Kolb’s view, however, the learner proceeds linearly around the stages, which is at odds with a non-linear dynamic systems approach. This can be resolved by applying a non-linear approach to Kolb’s theory, in which the stages are maintained, but the pathway through them becomes more dynamic (see Figure 2).

Taken together, Kolb’s emphasis on learning as a cyclical process through which agents engage in concrete experience and the transformation of that experience aligns well with a dynamic systems and PSM approach to cognition, providing an explanatory framework for the ways in which engagement with the social and material environment leads to learning. On this model, learning is an ongoing process that continually cycles through the various stages in which no stage is developmentally superior to the others. Thus, an educational model that focuses on only one aspect of the cycle (for example, abstract conceptualization, as in much sedentary institutional education) is incomplete and does not properly facilitate learning. We see this problem at play in what has been dubbed the formalism first problem—when educational paradigms assume that knowledge can only be applied to real-world contexts when students have first mastered abstract concepts (Nathan, 2012). The Kolb cycle rejects the formalism first approach by showing that learning happens in recursive cycles that are in commerce with the environment. This theory aligns well with a 4E perspective in its attention to dynamic engagement with the social and material environment. However, Kolb proposes a linear progression through cycle, which does not account for the complex and often surprising interactions we experience as educators. We argue that applying a 4E lens to Kolb’s theory disrupts the linear nature of a learner’s pathway through the stages. Kolb’s theory also considers the learner as a, primarily, autonomous agent. Integrating PSM with Kolb would suggest, however, that the learner’s experience at each stage of the cycle is co-constructed and co-constituted by other agents, a point to which we will next turn our attention.

## 5 Putting it all together—group role formation and learning styles

**Figure 3:**
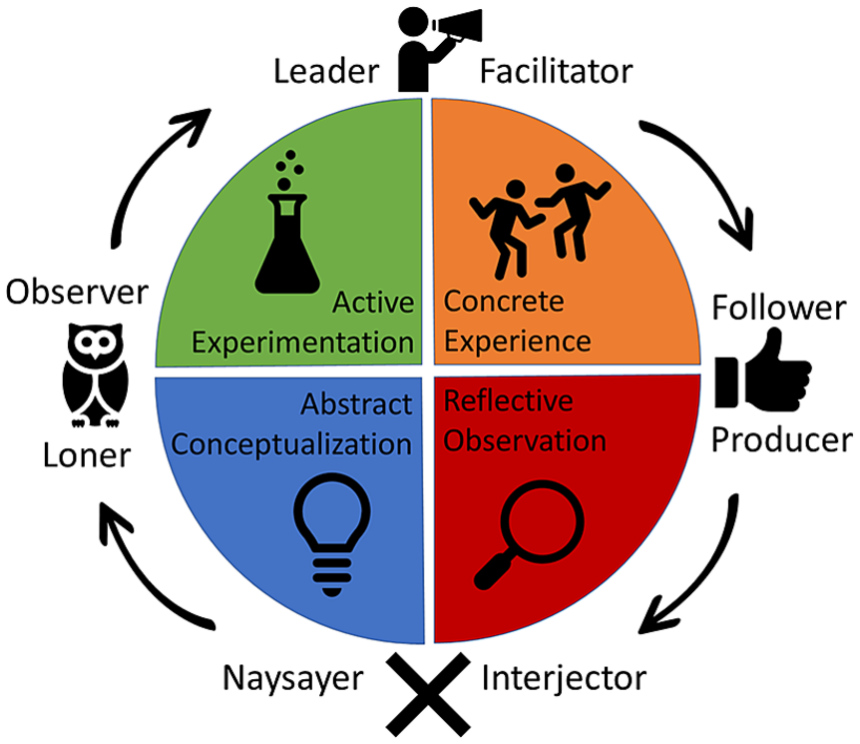
In this figure, we conceptualize how group roles and learning phases can cycle and become an emergent product of interaction. A naysayer, in their interaction with the group, might move from reflective observation through abstract conceptualization and into active experimentation. In doing so, as an emergent product of interaction, the naysayer will likely switch roles, moving from the naysayer through the observer role to the facilitator role.

Calling on PSM we have argued that the cognitive grappling that occurs when more than one agent is present is dynamic, emergent, and cannot be reduced to individuals’ cognitive activity. Placing PSM in a pedagogically directed environment, we draw on Kolb’s Learning Cycles to highlight how group thinking and learning involve a non-linear cycling through a series of modes of engagement. To these group interactions, learners bring their own histories, manifested through skilled habits (Bruineberg et al., 2021). According to Kolb, individuals have a preference for specifically defined learning styles that relate to the learning cycle: Accommodating, Diverging, Converging, and Assimilating. These styles are related to the learning cycle by conceiving of the four stages of the cycle as divided by two axes (see Figure 3), one representing thinking and feeling and the other representing doing and watching. However, as will become clear as we develop our embodied and enactive take on the learning cycle, thinking, feeling, action, and observation are all intermingled and can only be separated as a philosophical abstraction.

Each individual in a learning group has a personal style of learning. The literature on group roles and learning has primarily focused on how scripted group roles can improve the overall productivity outcome of groups; only a sparse number of studies focus on the dynamics of emergent group roles (see He et al., 2023 for lit review). However, from this empirical literature emerges a consistent pattern;—human group members tend to fall into one of four overarching group role categories particularly in the embodied group creative paradigm (Giguere, 2024; Giguere, 2021; Pai et al., 2015).

1. The leader/facilitator/organizer (Exhibits leadership in the presentation of ideas and suggestions and directs group behavior)
2. The follower/producer (Participates actively in following suggestions presented to the group, but not as an initiator)
3. The naysayer/interjector (Rejects or presents counter arguments to suggestions made by leaders/facilitator/organizer)
4. The loner/observer (Works separately from the group, but returns to the group to make suggestions, follow directions or express reactions at points during the creative process)

Given the emergent nature of PSM interactions each member will be causally impacted by the group itself to cycle through new group roles and new sections of the learning cycle. These group roles are emergent instead of scripted; the roles develop from interactions between group members rather than being formally given (Strijbos and De Laat, 2010). Once in place, emergent roles have temporary inertia and shape the future ways in which the agent engages with others (He et al., 2023). As summarized by Dowell et al. (2020) we identified that the roles participants occupy while engaging in problem-solving activities have distinct patterns in behavioral engagement style (i.e., active or passive, leading or following). Some of the most noteworthy discoveries concern the influence of roles on student learning and team performance, which suggests that the difference in outcome measures across the roles is not a result of individuals simply being more active in terms of a number of contributions. The quality of conversation, as captured by the internal cohesion, responsivity, and social impact measures, more than the quantity, appears to be the key element in the success for both teams and individuals during problem-solving activities (Dowell et al., 2020, p. 50–51).

Put differently, emergent roles are (just as we saw at the systems level with PSM) metastable modes of interaction that non-linearly impact the behavior of the whole system. In addition, as we see from Dowell et al. (2020) (and consistent with the Kolb cycle), group roles participate at different frequencies; however, it is the kind of contribution that matters. In this sense, passive members are not “worse” than active members. Rather, each role contributes essential styles of input to the progression of the overall system. Below, we integrate empirical literature on group learning, group roles, and leadership to argue that the roles described above can be mapped onto the different phases of Kolb’s learning cycle.

Group members in leadership roles tend to start in active experimentation. Given the current state of the system, group member skills, shared concepts, goals, and so on, leaders tend to organize others into different tasks. Put simply, the leader/facilitator tends to delegate tasks and take initiative to push the group in a new direction (active experimentation). For example, research on emergent group roles and communication patterns demonstrates that leaders tend to facilitate higher degrees of success across collaborative learning outcomes and push groups toward higher degrees of collaborative activity by using skills such as “maintaining communication, sharing ideas, regulating problem-solving, and negotiating ideas” (Dowell et al., 2020). Other studies conclude that emergent leader roles are often responsible for initiating cognitive engagement and concept construction (Ouyang and Chang, 2019). Such activity patterns are consistent with the active experimentation Kolb phase. Leaders utilize the group’s existing and new resources to facilitate new directions and explore novel results. In this way, leaders tend to be in the active experimentation phase of the Kolb cycle. In a dance choreography-making context, we often see one or two students taking the lead delegating sub-tasks. For example, a common comment we hear from students is “You make the first three eight-counts. We need to use the front pivots we learned in class. And can you make three eight-counts using back pivots?”

When leaders delegate, they also adjust and constrain the field of affordances available to other group members and temporarily stabilize the current emergent group roles of each group member. In this way, leaders/facilitators stabilize the overall system and its fields of affordances.

Followers often find themselves in the mode of concrete experience. Followers perform the tasks they have been delegated by gathering experience, resources, and information or producing specific products. In short, followers get things done. Historically, empirical research has assumed that leaders make things happen and that followers are simply empty vessels ready to be pushed into action (Kelley, 1992). However, followership is not simplistic; a nascent empirical literature on followership demonstrates that following is active, includes critical thinking, comes in sub-varieties, and that followers are often flexible and open to taking on new roles (Bastardoz and Van Vugt, 2019; Carsten et al., 2018; Matshoba-Ramuedzisi et al., 2022). The literature outlines how followership comes in different critical thinking varieties (both from independent action and as a response to specific leaders). Thus, followers often move through various learning phases to fulfill their required tasks (Utomo et al., 2022; Matshoba-Ramuedzisi et al., 2022). In particular, following specified tasks requires the application of critical thinking, filtering of unnecessary information, focused attention, and staying on task, (Carsten et al., 2018). In general, the role of the follower skews toward engaging with concrete experience because the task of following involves prolonged attention toward the same set of tasks, filtering unnecessary information from relevant information, and actively using the body in a task to create more experience.

Given their focus, followers also stabilize the group’s shared field of affordances. By engaging with concrete experience, followers tend to bring more resources or information into the system. We see then, that as followers work on assigned tasks they often increase the nesting of the affordance field (making affordances denser with more ecological information). However, because of their flexibility, followers can easily create or be impacted by phase transitions that will move them into other emergent group roles.

In group contexts, naysayers/interjectors tend to start in reflective observation and therefore interject themselves into established processes. The nay-sayer, often synthesizes, reflects, and re-evaluates the experiences or products created by leaders and followers (Giguere, 2021). Naysayers often provide a fresh new perspective on the progress of the group by rejecting the direction of specific processes—often those initiated by leaders (Giguere, 2011). By doing so, the naysayer is a crucial component in pushing the overall system through a phase transition into a different mode of engagement, often changing the emergent qualities of the group. While the label “naysayer” often has a negative connotation, the naysayer is a vital part of the group cognitive process (Giguere, 2024). A wealth of empirical research on improvisatory group dynamics demonstrate that groups as whole systems tend to move toward higher degrees of creativity and novelty when group members deliberately perturb the system out of its routine or current trajectory (Laroche et al., 2024). Naysaying is a form of perturbation that introduces novelty and shakes up the system; novel movements and interactions are produced during the perturbation and the re-stabilization of the system. In other words, much novelty in creative systems is generated in the oscillation between stability and instability created by the Naysayer. In fact, literature on emergent group roles and problem-solving sometimes redefines naysaying as a form of leadership (Ouyang and Chang, 2019). In the case of dance choreography, we often see a student opposing aesthetic choices made by leaders and followers. For example, “I do not think that move fits with the theme of this dance. We need something that looks more airy. Can we do more of a jump instead? Or maybe do this whole phrase a bit lighter on the feet, maybe on the and-counts?”

The naysayer is often an important interlocutor for novelty and creativity. Research has shown that creativity and novelty emerge from perturbations that push a creative system into phase transitions (Laroche et al., 2024). Similarly, recent research on creativity and embodiment, demonstrates that the creative use of affordances emerges from the introduction of constraints and obstacles (Feiten et al., 2023). For the internal dynamics of a group, the naysayer perturbs the system thereby creating different shared affordances. By perturbing the system the naysayer also often pushes the system into new phase transitions that further create affordances through constraints and pushes the emergence of new group roles. In this way, the naysayer is a crucial participant in the system’s internal dynamics.

Finally, in abstract conceptualization, we often find the loner/observer who typically takes a step back from the interactions of the group to gain a “big picture” view of the situation. Here, the term loner can often have a negative connotation. This group member creates generalizable principles given what the group has already done. The loner can easily be confused with a disengaged member because they often remove themselves from the immediate action of the situation. However, as research has shown the observer will often reassert themselves into the creative process at crucial junctures, perturbing the overall direction of the group with the realizations and products they have created when being away from the interactions of the group (Giguere, 2011; Dowell et al., 2020). Research has shown that “leadership” in groups is a more distributed feature in which “intellectual leadership” the task of conceptualizing, making conclusions, and drawing connections between concepts and evidence, sometimes falls on the group member taking a step back (Mercier et al., 2014). That is, the “loner” can be redefined as an active observer who is taking a form of intellectual leadership, interjecting at key moments (Dowell et al., 2020; Dowell et al., 2019). Intellectual contributions made by these group members can then be used by the facilitator to redirect tasks for each group member. The observer is often responsible for making sure that concepts are generated and understood amongst all group members. In dance making scenarios, we often see a student physically stepping back from the group to look at the rest of the group performing the choreography. This student might try to make overarching aesthetic conceptual changes to the choreography: “I think we need to look more moody, when we do that whole middle part.”

Taken together, the empirical literature on emergent group roles suggests that the starting points for the four standard group roles are consistently related to the different phases of the Kolb learning cycle. However, as we have seen so far (and the literature concurs), group roles and learning phases dynamically shift through interaction. When a group member takes on a particular role, for example the follower, that role often begins by corresponding to a specific Kolb learning phase, in this case, Concrete Experience. As interaction unfolds, the affordances provided by other group members will move the agent through learning phases until a critical phase transition pushes the agent into a new emergent group role. In one instance, the member in the follower role might transition to the leader role to engage a friend in the group who is in loner mode. Or the member in follower mode might transition to naysayer in response to a suggestion from a member in the leader role. Through interaction toward achieving the group’s goals, members frequently morph into holding different roles (Dowel et al., 2018). Group composition and group roles are dynamic and morph non-linearly, just as we saw with phases in the Kolb learning cycle. Group members can cycle through various roles, and the composition of any one group does not have to consist of only one of each (He et al., 2023). Rather, groups tend to be composed of different role combinations, which leads to different emergent dynamics (Giguere, 2021). For example, the composition of a group can be leader, leader, naysayer, naysayer, follower, observer.

**Figure 4:**
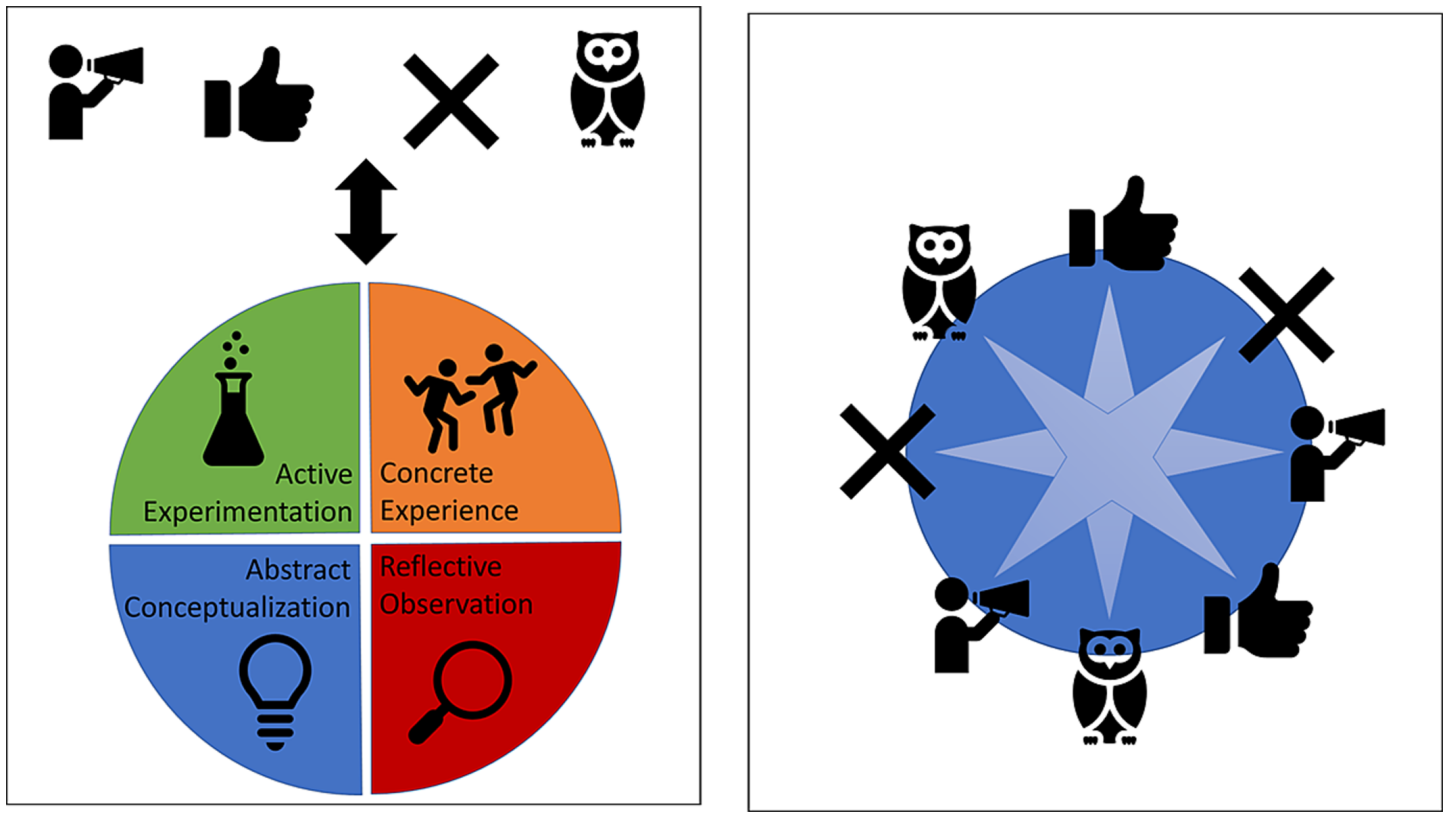
On the left is the bottom-up top-down causal relationship between learning phases and group roles. On the right is the emergent relationship between group members in different roles and learning phases and the collective field of affordances. Processes from the box on the left and those from the box on the right also influence each other bidirectionally.

Given the non-linear dynamic and emergent nature of human groups as participatory sensemaking systems, group roles and learning phases multi-directionally impact one another. The affordance space generated by group members, given the historicity of their interactions, produces system-internal perturbations so that group members in different roles begin moving through the various phases of the learning cycle. Often, as this happens, such a movement of phases will morph the composition of group roles so that group members will shift into new roles. This shifting of roles, in turn, reintroduces causal efficacy in group members’ learning phases. Group role compositions at the group level impact learning phases, and learning phases impact group roles (see Figure 4).

Through the ongoing multidirectional causal flow between learning phases and group roles a unique shared field of affordances emerges that provide group members with possibilities above and beyond the creative possibilities available to agents working alone. In fact, it could be argued that group interaction scaffolds learners into a rich engagement with different learning phases, often pushing them to spend more time within learning phases on which they would not ordinarily focus.

## 6 Future directions—groups as the unit of analysis

Through this conceptual analysis, we have argued that, as individual members cycle through various group roles and their associated Kolb phases, they aid the group as a joint cognitive system in transitioning to new modes of engagement at the group level. Moving through group roles (leader, follower, naysayer, observer) often moves the agent into a new Kolb phase, which, in turn, changes the emergent dynamics of the entire group. Thus, social interaction can drive the learning cycle. Because the behavior of the individual is dynamic and emergent, we cannot rely on reductivist accounts to explain group behaviors as the outcome of individual contributions. Rather, we consider the group, as a whole, as a cognitive agent in its own right. Individual agents causally influence the behavior of the group, but the group is simultaneously causally influencing the behavior of the individuals. Rather than positing testable descriptions or arguing for generalizable implications, our goal here is to develop a novel framework for an explicitly enactive theory of group learning. Future research will operationalize this framework by developing empirically testable descriptions of; (1) Changes in learning phases at both the individual and group level, (2) Changes in the collective field of affordances and how those map on to Kolb’s learning phases, (3) Changes in group roles and their relationship to PSM’s co-regulated coupling. Creation of such descriptions begin with careful qualitative analysis of in-person observation and video footage of young people engaged in collaborative dance-making in real-world classroom contexts. From this data testable hypotheses can be produced using the operationalized descriptions of points one, two, three, and four.

We suggest that collaborative dancemaking has several qualities that make it a productive site for examining the phenomenon of group thinking from a 4E lens. (1) In dancing and dance-making the thinking often happens in overt and observable ways. Therefore, the social dynamics, so critical to our understanding of the learning process, are transparent. (2) Dancing affords the opportunity to observe this phenomenon in an ecologically valid context, since dancemaking is naturally a group process without the need for an invented experimental structure. (3) Collaborative dance making is process-driven with multiple divergent solutions, affording the opportunity for each group to develop unique creative learning processes, as opposed to more linear problem solving.

Future examination of the embodied group creative process, using the methodological considerations listed above, would allow us to empirically describe and test our theoretical argument developed in this paper. If what we have argued holds true, then one implication is that the Kolb cycle, at least for group learning, is far less linear than proposed by Kolb. The dynamic interactions could scaffold the group moving directly from concrete experience into abstract conceptualization or from reflective observation into active experimentation. Considering the non-linear and disruptive function of group social engagement, a non-linear progression through the Kolb cycle seems possible, even likely. Given our contention that individual learners’ paths are non-linear and that the individual’s social role energizes other learners into less expected ways of interacting with the learning process, we would also expect to see multiple groups arrive at their learning outcomes in multiple ways. This could be evidenced by two groups with similar composition, doing the same task, yet arriving at different results or similar results in vastly different ways.

The critical implications of this analysis are twofold: the social interactions that are integral to fluid social role formation are the affordances that power the experiential learning cycle. Students flow from one stage of Kolb’s cycle to another because of the impact of social engagement. We cannot understand the learning cycle of each individual in isolation because their educational experience intersects with the social dynamics of the learning environment. To have a more complex understanding of children’s learning, therefore, we need to consider cognitive grappling at the group level. Applied to an empirical research agenda, several methodological considerations emerge:

- How can the learning stage of a group, as a whole, be empirically evidenced?
- How can transitions in learning stages of the group be empirically evidenced?
- How can the bi-direction causal relationships between a group’s learning stage and the learning stages of individual members be evidenced?
- Further, how can this be evidenced in a way that respects the dynamism and non-linearity at the level of both individuals and groups?

In the introduction to this paper, we suggest, based on our experiences as dance educators, when groups get together to make a dance that each group takes on a life of its own, one that cannot be predicted by understanding only the habits and dispositions of its individual members. Through the subsequent analysis we have constructed an argument, based on 4E cognitive theory and empirical evidence, that provides an explanatory framework for this pedagogical observation. As group members cycle through various roles (leader, follower, naysayer, loner), they move the system through phase transitions into new modes of engagement. Their interactions give rise to non-linear emergent interactions at the group level, which in return has a causal impact on all group members’ cognitive activity; a form of bi-directional bottom-up-top-down causation.

It is our intention to conduct future research into experiential learning across contexts, which allows for not only testing of this theoretical argument, but also an openness to new discoveries into the complexities of group learning, which prioritizing the group as the unit of analysis might allow. Although our current focus is on creative learning settings, namely dance-making, recent literature in activity-based learning strongly suggests that our proposed framework could be generalizable to other learning contexts (Kronsted, 2025; Gautam and Kumar, 2020; Becker, 2023). In a broader sense, we propose that, when viewed through a dynamic and emergent lens, collaborative dancemaking is a productive site for understanding group learning beyond the dance context; in short, looking at dance teaches us about minds in general.

## Data Availability

The original contributions presented in the study are included in the article/supplementary material, further inquiries can be directed to the corresponding authors.
